# Deletion of the FHL2 gene attenuates intima‐media thickening in a partially ligated carotid artery ligated mouse model

**DOI:** 10.1111/jcmm.14687

**Published:** 2019-11-12

**Authors:** Chi‐Yu Chen, Hsiao‐Ya Tsai, Shih‐Hung Tsai, Pao‐Hsien Chu, Po‐Hsun Huang, Jaw‐Wen Chen, Shing‐Jong Lin

**Affiliations:** ^1^ Institute of Clinical Medicine National Yang‐Ming University Taipei Taiwan; ^2^ Department of Emergency Medicine Tri‐Service General Hospital National Defense Medical Center Taipei Taiwan; ^3^ First Division of Cardiology Department of Internal Medicine Chang Gung Memorial Hospital Chang Gung University College of Medicine Taipei Taiwan; ^4^ Division of Cardiology Taipei Veterans General Hospital Taipei Taiwan; ^5^ Department of Critical Care Medicine Taipei Veterans General Hospital Taipei Taiwan; ^6^ Cardiovascular Research Center National Yang‐Ming University Taipei Taiwan; ^7^ Department of Medical Research Taipei Veterans General Hospital Taipei Taiwan; ^8^ Institute and Department of Pharmacology National Yang‐Ming University Taipei Taiwan; ^9^ Healthcare and Management Center Taipei Veterans General Hospital Taipei Taiwan; ^10^ Taipei Heart Institute Taipei Medical University Taipei Taiwan

**Keywords:** carotid artery ligation, FHL2, neointimal hyperplasia, smooth muscle cell

## Abstract

The four and a half LIM domain protein 2 (FHL2) is a member of the four and a half LIM domain (FHL) gene family, and it is associated with cholesterol‐enriched diet‐promoted atherosclerosis. However, the effect of FHL2 protein on vascular remodelling in response to hemodynamic alterations remains unclear. Here, we investigated the role of FHL2 in a model of restricted blood flow‐induced atherosclerosis. To promote neointimal hyperplasia in vivo, we subjected FHL2^+/+^ and FHL2^−/−^ mice to partial ligation of the left carotid artery (LCA). The expression of p‐ERK and p‐AKT was decreased in FHL2^−/−^ mice. FHL2 bound to AKT regulated AKT phosphorylation and led to Rac1‐GTP inactivation. FHL2 silencing in human aortic smooth muscle cells down‐regulated the PDGF‐induced phosphorylation of ERK and AKT. Furthermore, FHL2 silencing reduced cytoskeleton conformational changes and caused cell cycle arrest. We concluded that FHL2 is essential for the regulation of arterial smooth muscle cell function. FHL2 modulates proliferation and migration via mitogen‐activated protein kinase (MAPK) and PI3K‐AKT signalling, leading to arterial wall thickening and thus neointimal hyperplasia.

## 1 INTRODUCTION

Vascular smooth muscle cell (VSMC) activation and phenotypic switching from the quiescent contractile state to the active synthetic state are critical for remodelling processes in vascular proliferative disorders,^1^, ^2^ including atherosclerosis, postangioplasty restenosis and vein bypass graft failure. Atherosclerosis with intimal injury affects various cell types, including endothelial cells (ECs), platelets and inflammatory cells, which release mediators such as growth factors and cytokines.^2^ Excessive neointimal hyperplasia after percutaneous transluminal angioplasty induces restenosis, which often necessitates a secondary revascularization procedure. During neointimal hyperplasia, VSMCs proliferate in response to mitogenic stimuli and migrate towards the luminal side of the artery.^3^ Both the migratory and proliferative activities of VSMCs, as well as the interplay between the extracellular matrix (ECM) and integrin receptors, contribute to neointimal hyperplasia and vascular remodelling in the vessels.^4^, ^5^ Although percutaneous transluminal coronary angioplasty and drug‐eluting stents are widely used, high rates (>10%) of in‐stent restenosis in clinical practice limit the treatments.^6^, ^7^ Given the high clinical relevance of proliferative vascular diseases, new and selective treatment strategies require a detailed understanding of the signalling mechanisms involved in VSMC activation.

Four and a half LIM domains 2 (FHL2) is a member of the four and a half LIM domain (FHL) family.^8^ The LIM domains are double zinc finger motifs that play multiple roles in protein‐protein interactions. For example, LIM can act as a scaffold in functional modifiers and adaptors.^9^ FHL2 is a LIM domain‐only protein that participates in cell transcription and signal transduction. It functions as a coactivator of several transcription factors, such as β‐catenin, AP‐1 and CREB,^10^ and interacts with myocardin family SRF cofactors.^11^ FHL2 modulates BMP‐mediated VSMC phenotypic switching.^12^ FHL2 regulates the inflammatory response by decreasing pro‐inflammatory monocyte recruitment and macrophage content.^13^ FHL2 plays a critical role in various physiological and pathological processes, including proliferation and migration.^14^ Although the role of FHL2 has been investigated in cardiovascular diseases in the past decade,^15^, ^16^ the relationship between FHL2 and neointimal formation remains controversial.

Our data indicate that inhibition of FHL2 inhibits cell proliferation by diminishing the phosphorylation of the downstream signalling molecules MEK1 and ERK1/2 in vitro and in vivo, thus causing cell cycle arrest. Furthermore, the results show that inhibition of FHL2 modulates cell migration by attenuating lamellipodia formation and inhibiting Rac1‐GTP activity. These data conclusively show that the tightly controlled down‐regulation of FHL2 represents an essential prerequisite for the proliferative response of VSMCs following vascular injury.

## 2 MATERIALS AND METHODS

### 2.1 Experimental animals

FHL2‐null mice (FHL2^−/−^) were provided by Dr Chu's laboratory. The endogenous ATG start codon of FHL2 was replaced by cDNA encoding LacZ and pGKneo cassettes. In this manner, the LacZ cDNA was brought under the control of the endogenous FHL2 promoter, thus ablating the expression of the endogenous FHL2 gene. All animals used in this study (mice homozygous null for the FHL2 gene and wild‐type littermates) had a C57BL/6 genetic background and were genotyped by PCR. All mice were kept in microisolator cages on a 12‐hour day/night cycle. All experimental procedures and protocols involving animals were approved and monitored by the Institutional Animal Care and Use Committee of Taipei Veterans General Hospital (IACUC no. 2014‐221, Taipei, Taiwan) and complied with the Guide for the Care and Use of Laboratory Animals published by the US National Institute of Health (8th edition, 2011).

### 2.2 Carotid artery ligation model

**Figure 1:**
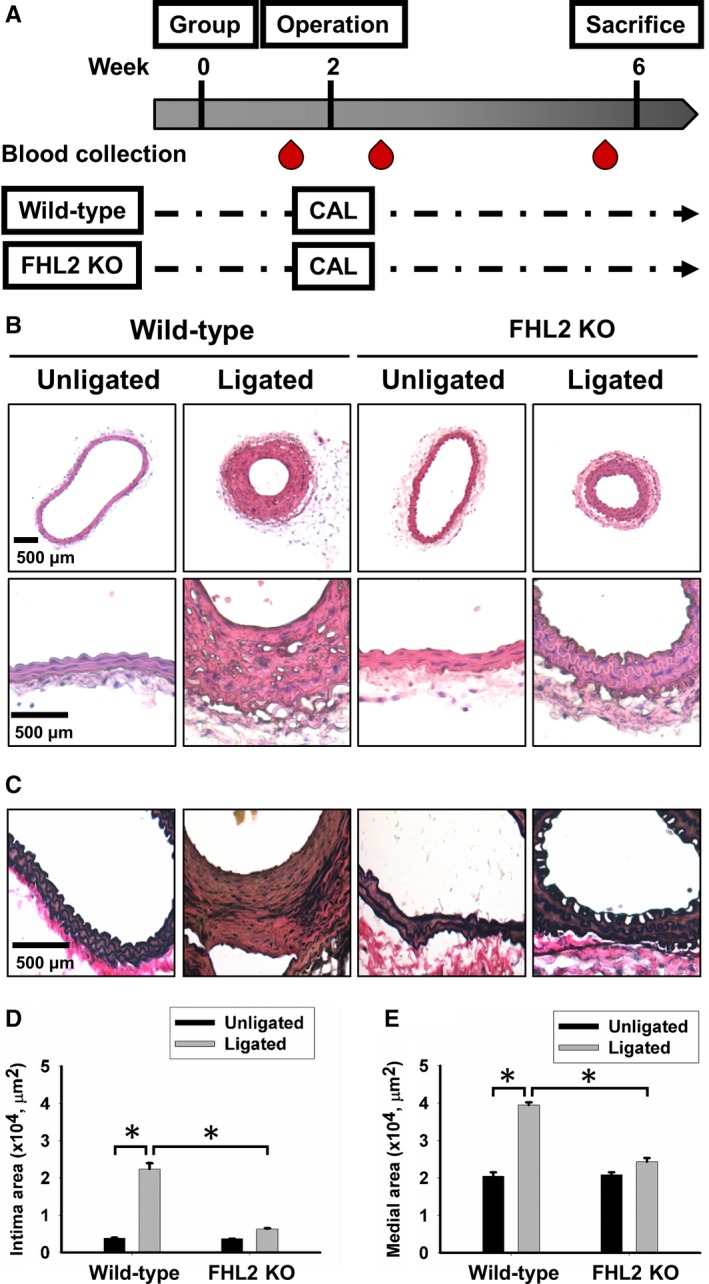
Lack of FHL2 retards neointima lesion formation. A, The workflow of animal study. Representative photomicrographs showing cross‐sectional areas (at 0.8‐1 mm from bifurcation) of the ligated carotid artery of wild‐type (FHL2^+/+^, C57Bl/6) and FHL2‐deficient (FHL2^−/−^) mice isolated 28 d after ligation (N = 10). Scale bars: 500 μm, (B) haematoxylin and eosin stain and (C) Verhoeff‐Van Gieson Stain images of cross‐sections from carotid arteries harvested 28 d after carotid ligation in FHL2^+/+^ and FHL2^−/−^ mice. D, The quantitative results of intima and (E) medial area were measured by the ImageJ software (NIH Image software). Bars represent average ± SEM of intimal and medial area. (*P < .01)

To induce neointimal hyperplasia in vivo, we subjected FHL2^+/+^ and FHL2^−/−^ mice to partial ligation of the left carotid artery (LCA). Mice were anesthetized with an intraperitoneal injection of 250 mg/kg avertin. Mice were considered to be adequately anesthetized when no attempt to withdraw the limb after pressure was observed. The LCA was exposed through a small midline incision in the neck. Partial ligation of the LCA was performed by ligating three branches of the LCA with a 6‐0 silk suture, including the external carotid artery (ECA), internal carotid artery (ICA) and occipital artery (OCA) while leaving the superior thyroid artery (STA) open. In all groups, the contralateral right common carotid artery (RCA) was left unligated and served as a control.^17^ At different time‐points, we isolated the ligated and contralateral unligated arteries for morphometric and Western blot analyses. The schema of the protocol was shown in Figure 1A.

### 2.3 Morphometric analysis

Four weeks after carotid artery ligation (CAL), the mice were euthanized by an intraperitoneal injection of avertin. The left ventricle was cannulated and perfused with phosphate buffer saline (PBS) and fixed with 4% paraformaldehyde (PFA). The left and right carotid arteries were collected and incubated in 4% PFA for 8 hours. After the fixed arteries were subjected to cryopreservation in 30% sucrose/PBS at 4°C, they were embedded in an optimal cutting temperature (OCT) compound and frozen. Cross‐sections (5‐µm thick) were taken starting at the carotid ligation site and were processed for haematoxylin and eosin (H&E) staining. Cross‐sections taken 1.5 mm proximal to the ligation site were obtained from each mouse. Four different regions (lumen, intima, media and total vascular area) of H&E stained cross‐sections were analysed using ImageJ software (NIH Image software). The areas surrounded by the luminal surface, internal elastic lamina and external elastic lamina were calculated. The intimal area was determined by subtracting the luminal area from the area defined by the internal elastic lamina, and the medial area was calculated by subtracting the area defined by the internal elastic lamina from the area defined by the external elastic lamina.

### 2.4 Cell culture

Three batches of human aortic smooth muscle cells (HASMCs) were purchased from Life Technologies and ScienCell. Human aortic smooth muscle cells were used at passage 4‐8. Cells were cultured in M231 medium supplemented with smooth muscle growth supplement (SMGS) in a humidified incubator (95% air and 5% CO2) at 37°C.

### 2.5 Transfection of siRNA

Human aortic smooth muscle cells were transfected with a FHL2‐specific siRNA (siFHL2) (10 nmol/L; Santa Cruz Biotechnology) or with scrambled siRNA (siControl) (Santa Cruz Biotechnology) as a transfection control for 24 hours using the Lipofectamine LTX transfection reagent (Life Technologies) in M231 medium containing SMGS.

### 2.6 Western blot

Cells were lysed in buffer (62.5 mmol/L Tris‐HCl, 2% SDS, 10% glycerol, 0.5 mmol/L PMSF and 2 µg/mL aprotinin, pepstatin and leupeptin), and the protein lysates were subjected to SDS‐PAGE followed by electroblotting onto a polyvinylidene difluoride (PVDF) membrane. Membranes were probed with monoclonal antibodies against FHL2 (1:500; Santa Cruz Biotechnology), *p*‐RAF1 (1:1000; Abcam), MEK1 (1:1000; Abcam), *p*‐MEK1 (1:1000; Abcam), ERK (1:1000; Abcam), *p*‐ERK (1:1000; Abcam), p38 (1:1000; Cell Signaling Technology), *p*‐p38 (1:1000; Cell Signaling Technology), Cyclin‐dependent kinase 2 (CDK2) (1:1000; Santa Cruz Biotechnology), Cyclin‐dependent kinase 4 (CDK4) (1:1000; Santa Cruz Biotechnology), cyclin E (1:1000; Santa Cruz Biotechnology), AKT (1:1000; Cell Signaling Technology), *p*‐AKT (1:1000; Cell Signaling Technology), PI3K p110β (1:1000; Abcam), Rac1 (1:1000; Abcam) and β‐actin (1:5000). Protein expression was visualized using chemiluminescence detection reagents (Merck Millipore). The results were analysed using ImageJ software.

### 2.7 Cell proliferation assay

The proliferative ability of HASMCs was determined using a Cell Counting Kit‐8 (CCK‐8; Sigma‐Aldrich). Human aortic smooth muscle cells were initially seeded at a density of 2 × 10^4^ cells per well on a 24‐well plate. After they were incubated with FHL2 siRNA and scrambled siRNA, the cells were cultured in M231 with or without 10 ng/mL of PDGF‐BB (Sigma‐Aldrich). Finally, the media were replaced with M231 containing 10X dilution of CCK‐8, and the cells were incubated for 4 hours for the proliferation assay. The absorbance was measured at 450 nm.

### 2.8 Cell migration assay

The migratory ability of HASMCs was evaluated using a modified Boyden chamber assay. Human aortic smooth muscle cells were dissociated with trypsin/EDTA, and the M231 medium containing 1 × 10^4^ HASMCs was placed in the upper chamber of a 24‐well transwell plate with polycarbonate membranes (8‐µm pores; Merck Millipore). M231 medium containing PDGF (10 ng/mL) was placed in the lower chamber as the attractant. After incubating for 8 hours, the membrane was briefly washed with PBS and fixed with 4% paraformaldehyde. The membrane was stained using haematoxylin solution, and the cells on the upper surface of the membrane were carefully removed. The magnitude of HASMC migration was evaluated by counting the migrated cells in six randomly chosen high‐power (X100) microscopic fields.

### 2.9 Cell cycle analysis

Cells were scraped and fixed with 70% ethanol 24 hours after treatment. Following RNase A treatment and propidium iodide (PI) staining, the DNA content in the cells was analysed using a Cytomics FC500 cell analyzer (Beckman Coulter). The distribution of at least 10 000 PI‐positive cells was analysed at different phases of the cell cycle using the Multicycle software.

### 2.10 Lamellipodia formation assay

To study lamellipodia formation, HASMCs were cultured to subconfluence on collagen‐coated glass coverslips. After 24 hours of starvation, the cells were treated with or without 10 ng/mL PDGF for 15 minutes. The cells were fixed for 5 minutes with cold methanol and immunostained with F‐actin. We quantified lamellipodia using fluorescence microscope images and gave scores of 0 and 1 to negative and positive cells, respectively.

### 2.11 Immunofluorescence

Carotid artery sections were blocked with bovine serum albumin (BSA) for 30 minutes and incubated with anti‐FHL2 (1:50; Santa Cruz Biotechnology) and anti‐αSMA (1:200; Abcam) antibodies for 16 hours at 4°C. Antibody staining was detected with Alexa Fluor‐555‐conjugated anti‐rabbit and Alexa Fluor‐488‐conjugated antimouse secondary antibodies (1:500; Life Technologies). Sections were stained with 4′,6‐diamidino‐2‐phenylindole (DAPI) as a counterstain, and the immunocytochemistry images were acquired using a Leica‐SP5 confocal microscope.

### 2.12 Co‐immunoprecipitation

The transfected cell lysates were co‐immunoprecipitated using a Pierce Co‐Immunoprecipitation Kit (Thermo Fisher Scientific). In short, detergent‐treated extracts of smooth muscle cells were cleared by centrifugation (5 minutes, 13 000 *g*) and then pre‐incubated (2 hours, 4°C) with protein G Sepharose and anti‐FHL2‐agarose beads. The beads were washed in lysis buffer, and the bound proteins were eluted by boiling in 2X sample buffer. The precipitated complexes were analysed by Western blotting with anti‐AKT or anti‐PI3K p110β antibody.

### 2.13 Rac1 activity assay

Rac1 activation was evaluated by detecting the GST‐CRIB binding domain on the activated form of Rac1 (Rac1‐GTP). Human aortic smooth muscle cells were cultured to subconfluence on culture Petri dishes in M231 medium. After 24 hours of starvation, the cells were treated for 5 minutes with normal medium (unstimulated) or medium containing PDGF (10 ng/mL; stimulated) in the presence or absence of FHL2 siRNA. Cell lysates (obtained as described above) were incubated with 4 µg of GST‐CRIB beads for 30 minutes at 4°C; then, the sample was washed, and bound Rac1 was eluted by boiling the samples in Laemmli buffer. Samples were separated on 15% SDS‐polyacrylamide gels, immunoblotted with a monoclonal anti‐Rac1 antibody (1:1000; Abcam) and developed with antimouse secondary antibody. Rac1 activation was determined by the ratio of the bands detected in the immunoprecipitants with the total Rac1 protein band present in the initial cell lysate.

### 2.14 Statistical analysis

Quantitative data are expressed as the mean ± standard error of the mean (SEM). Comparisons between two groups were performed using unpaired Student's *t* test and multiple group comparisons were performed using one‐way ANOVA followed by Scheffe's multiple‐comparison post hoc test. Data were analysed using SPSS software (version 14; SPSS). A *P* value of <.05 was considered to indicate statistical significance.

## 3 RESULTS

### 3.1 FHL2 deficiency attenuates neointimal formation in mice

**Figure 2:**
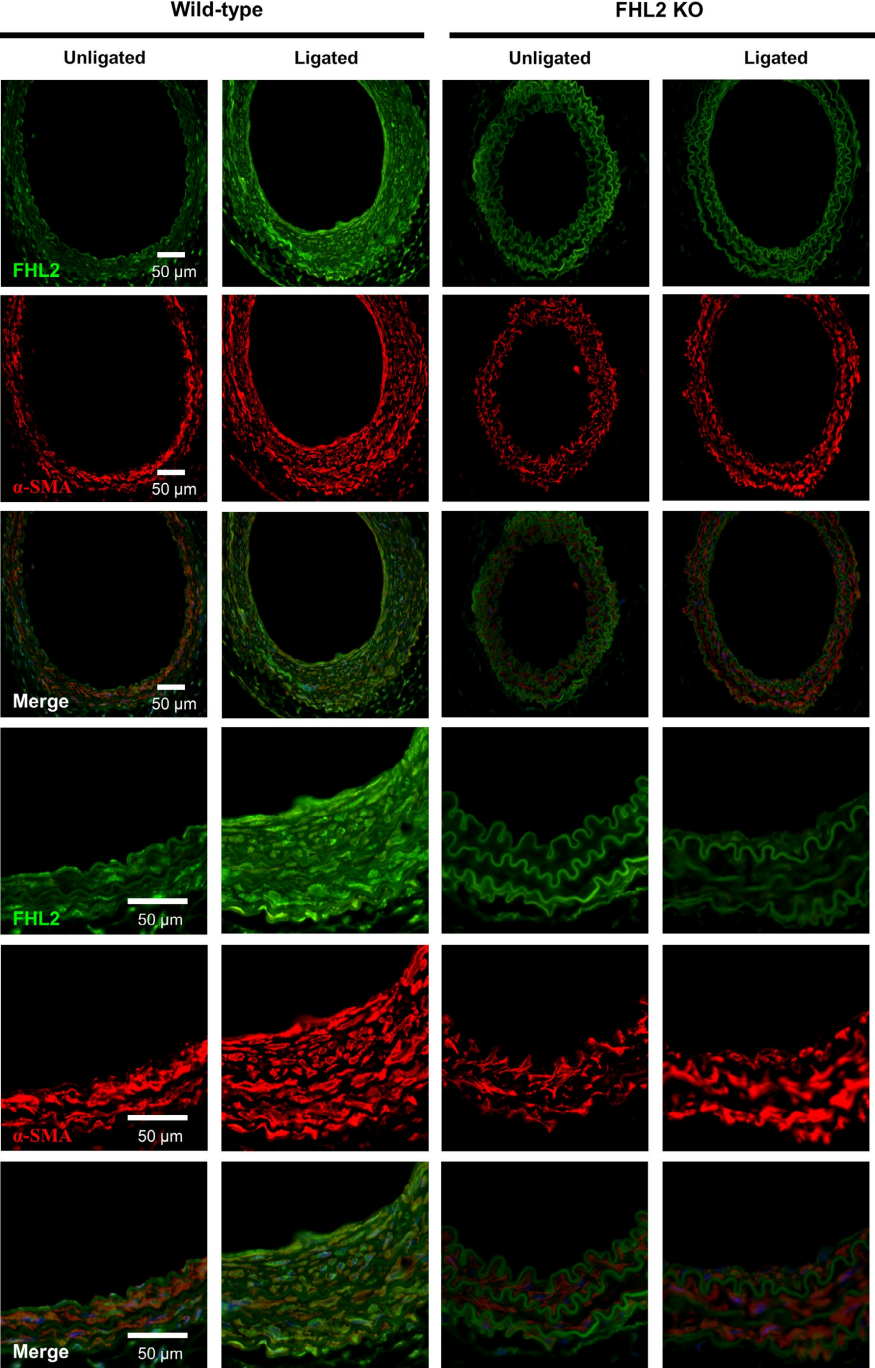
Expression of FHL2 in arterial SMCs after ligation. Neointimal formation was induced by ligation of left carotid artery of FHL2^+/+^ and FHL2^−/−^ mice. Immunofluorescence analysis (28 d after ligation) showed co‐localization of FHL2 with αSMA^+^ cells (n = 10). The common carotid arteries from mice were fixed with 4% formaldehyde and cut into 7 μm frozen sections. Immunostaining for FHL2 (green), α‐SMA (red) and Hoechst (blue)

We examined the effect of FHL2 deficiency on neointimal hyperplasia in vivo. There were no morphological differences between the contralateral carotid arteries of FHL2^+/+^ and FHL2^−/−^ mice (n = 10; Figure 1B). As expected, 4 weeks after ligation, obvious neointimal and medial hyperplasia could be observed in FHL2^+/+^ mice, whereas neointimal thickening was less pronounced in FHL2^−/−^ mice than in FHL2^+/+^ mice (n = 10 in each group; Figure 1B,C). The quantitative data also demonstrated that the intimal cross‐sectional area was smaller in FHL2^−/−^ mice than in FHL2^+/+^ mice (n = 10 in each group; Figure 1D,E). Consistent with a previous study, neointimal hyperplasia reached a maximum at approximately 4 weeks after ligation in FHL2^+/+^ mice. Interestingly, the effects of neointimal hyperplasia were decreased in FHL2^−/−^ mice at 4 weeks after ligation (n = 10 in each group; Figure 1D,E). Furthermore, immunofluorescence staining with an anti‐FHL2 antibody revealed that FHL2 expression significantly increased in the area of intimal thickening and in the medial layer in FHL2^+/+^ mice compared with in FHL2^−/−^ mice (n = 10 in each group; Figure 2).

### 3.2 FHL2 deficiency inhibits proliferation signals and cytokine secretion in vivo

**Figure 3:**
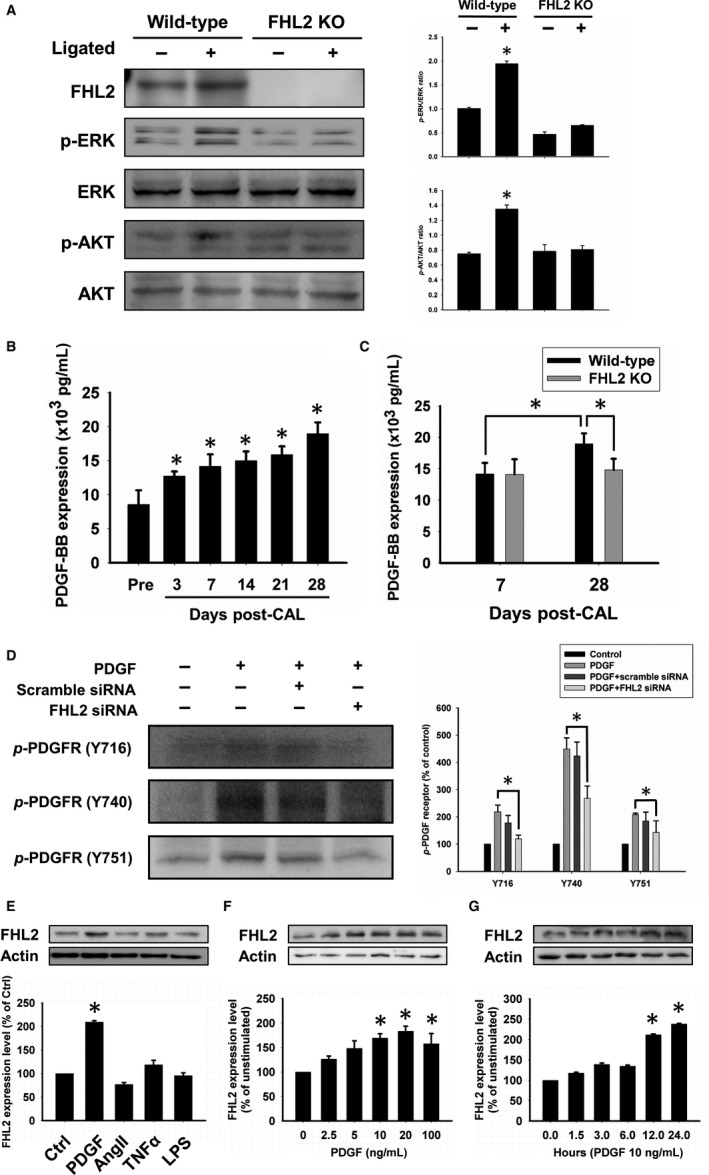
Expression of ERK and AKT of the artery of the mice, and the secretion level of PDGF after carotid ligation. The vascular injury was induced by ligation of the carotid artery in mice. Arteries were collected 2 wk after ligation. A, The ratio of ERK and AKT phosphorylation was analysed using Western blotting. B, Serum was collected from wild‐type mice in different time‐points after carotid artery ligation. The level of PDGF secretion was measured using ELISA. C, The comparison of PDGF level in FHL2^+/+^ and FHL2^−/−^ mice. The results were expressed as the mean ± SEM of five separate experiments run in triplicate. (*P < .01 vs before ligation) (D) HASMCs were treated with PDGF for 24 h. Knockdown of FHL2 decreased the level of PDGF receptor phosphorylation. E, HASMCs were treated with a series of cytokines for 24 h. PDGF significantly increased the level of FHL2. FHL2 protein expression up‐regulated in a dose and time‐dependent manners (F, G) after PDGF treatment. These results are expressed as the mean ± SEM of five separate experiments run in triplicate. (*P < .01 vs unstimulated cells)

Western blot data showed that the expression level of FHL2 significantly increased after carotid artery ligation (CAL) in FHL2^+/+^ mice and that the expression level of FHL2 was unchanged after CAL in the FHL2^−/−^ mice (n = 10 in each group; Figure 3A). Furthermore, CAL significantly increased the phosphorylation of ERK and AKT in FHL2^+/+^ mice but not in FHL2^−/−^ mice (n = 10 in each group; Figure 3A).

Platelet‐derived growth factor (PDGF) plays an essential role in vascular remodelling. To determine the level of PDGF during vascular remodelling, serum was collected from FHL2^+/+^ after carotid ligation at different time‐points. The ELISA data showed that the level of PDGF increased with time (n = 10 in each group; Figure 3B). The level of PDGF was higher in FHL2^+/+^ mice than in FHL2^−/−^ mice at 4 weeks after carotid ligation (n = 10 in each group; Figure 3C).

### 3.3 PDGF regulates FHL2 expression

Result showed that knockdown of FHL2 decreased the phosphorylation of PDGF receptor (n = 5; Figure 3D). To determine whether PDGF is a major cytokine in FHL2 induction, human aortic smooth muscle cells (HASMCs) were treated with a series of inducers for 24 hours. Compared to stimulation with other cytokines, PDGF‐induced significant overexpression of FHL2 (n = 5; Figure 3E). Furthermore, PDGF dose‐ and time‐dependently up‐regulated the expression of FHL2 in HASMCs (n = 5; Figure 3F,G).

### 3.4 Knockdown of FHL2 expression inhibits cell proliferation and signalling in HASMCs

**Figure 4:**
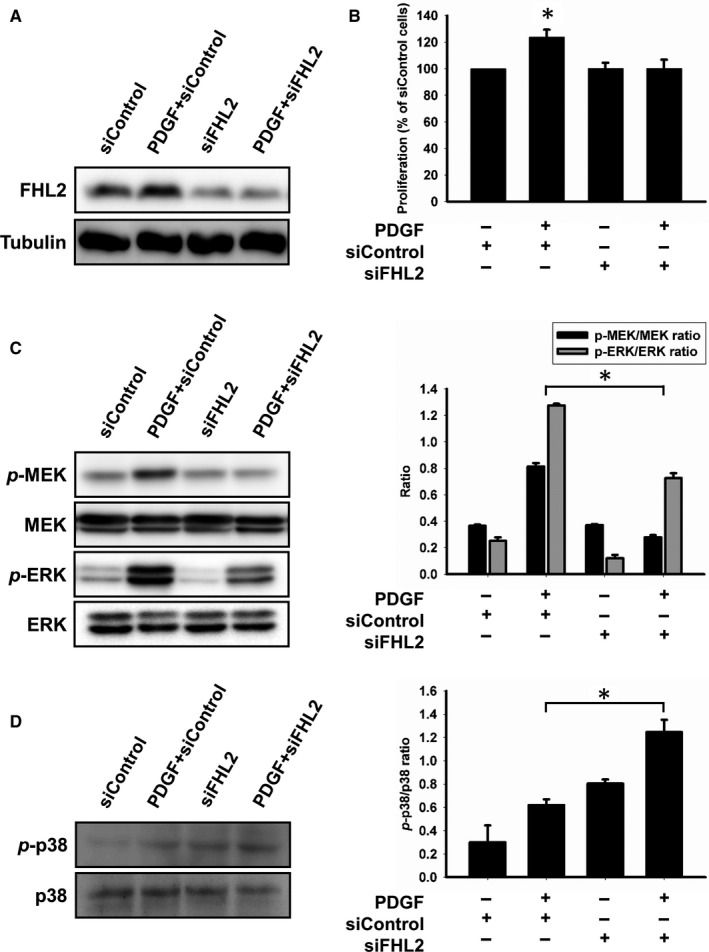
Effect of FHL2 on the signalling mediated the phosphorylation of MEK, ERK and p38. FHL2 deficiency modulated cell proliferation ability and the MAPK signalling pathway. A, FHL2 siRNA inhibited the FHL2 protein expression in HASMCs after 24 h transfection. B, Cell proliferation of transfected HASMCs evaluated by a Cell Counting Kit‐8. HASMCs were transfected with the control or FHL2 siRNA. After 24 h, cells were stimulated with PDGF for 15 min, MEK/ERK (C) and p38 (D) phosphorylation were analysed using Western blotting. These results are expressed as the mean ± SEM of five separate experiments run in triplicate. (*P < .01)

Knockdown of FHL2 expression was achieved via the transfection of FHL2 siRNA. FHL2 silencing repressed the expression of FHL2 by up to 90% (n = 5; Figure 4A) and significantly inhibited PDGF‐induced HASMC proliferation (n = 5; Figure 4B). The mitogen‐activated protein kinase (MAPK) signalling pathway is important for cell proliferation, and FHL2 silencing inhibited the PDGF‐induced phosphorylation of MEK and ERK (n = 5; Figure 4C). In contrast, FHL2 silencing induced p38 phosphorylation (n = 5; Figure 4D).

### 3.5 Knockdown of FHL2 expression regulates cell cycle arrest

**Figure 5:**
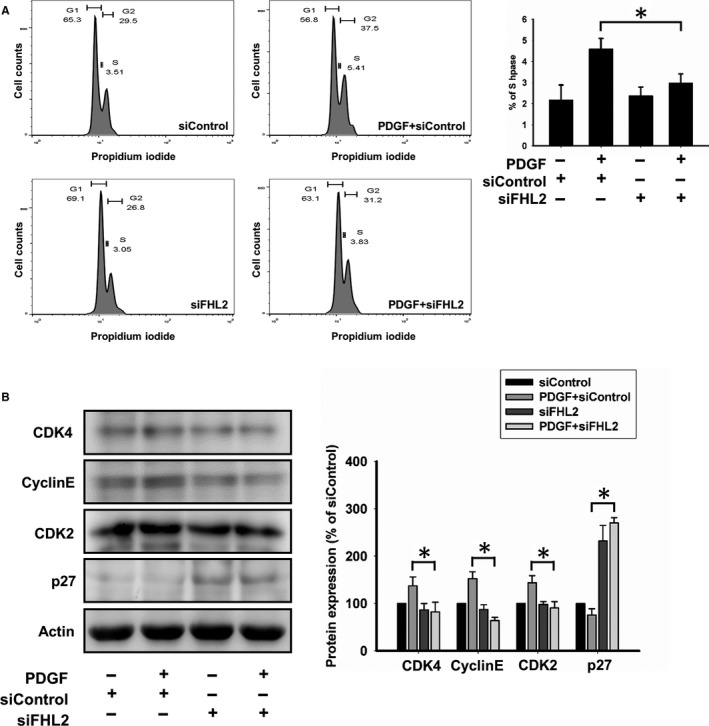
FHL2 deficiency induced G1 cell cycle arrest in HASMCs. A, HASMCs were transfected with FHL2 siRNA and serum starved for 24 h. Cells were stimulated with PDGF for 24 h, and the cell cycle distribution was analysed using flow cytometry and expressed as a percentage of cells in the S phase. B, HASMCs transfected FHL2 siRNA and serum starved for 24 h. Cells were stimulated with PDGF for the indicated time, and Western blot analysis for cell cycle‐regulatory proteins was performed. These results are expressed as the mean ± SEM of five separate experiments run in triplicate. (*P < .01)

The inhibition of cell proliferation was correlated with the induction of cell cycle arrest in FHL2 knockdown cells. FHL2 silencing reduced the number of HASMCs in the S phase (n = 5; Figure 5A). Moreover, FHL2 silencing decreased the expression of factors that promote the cell cycle, including cyclin E and cyclin‐dependent kinase (CDK) 2 and CDK4 and increased the expression level of the CDK inhibitor (CDKI) p27^kip1^ (n = 5; Figure 5B).

### 3.6 Knockdown of FHL2 expression inhibits cell migration and eliminates cytoskeleton conformational changes

**Figure 6:**
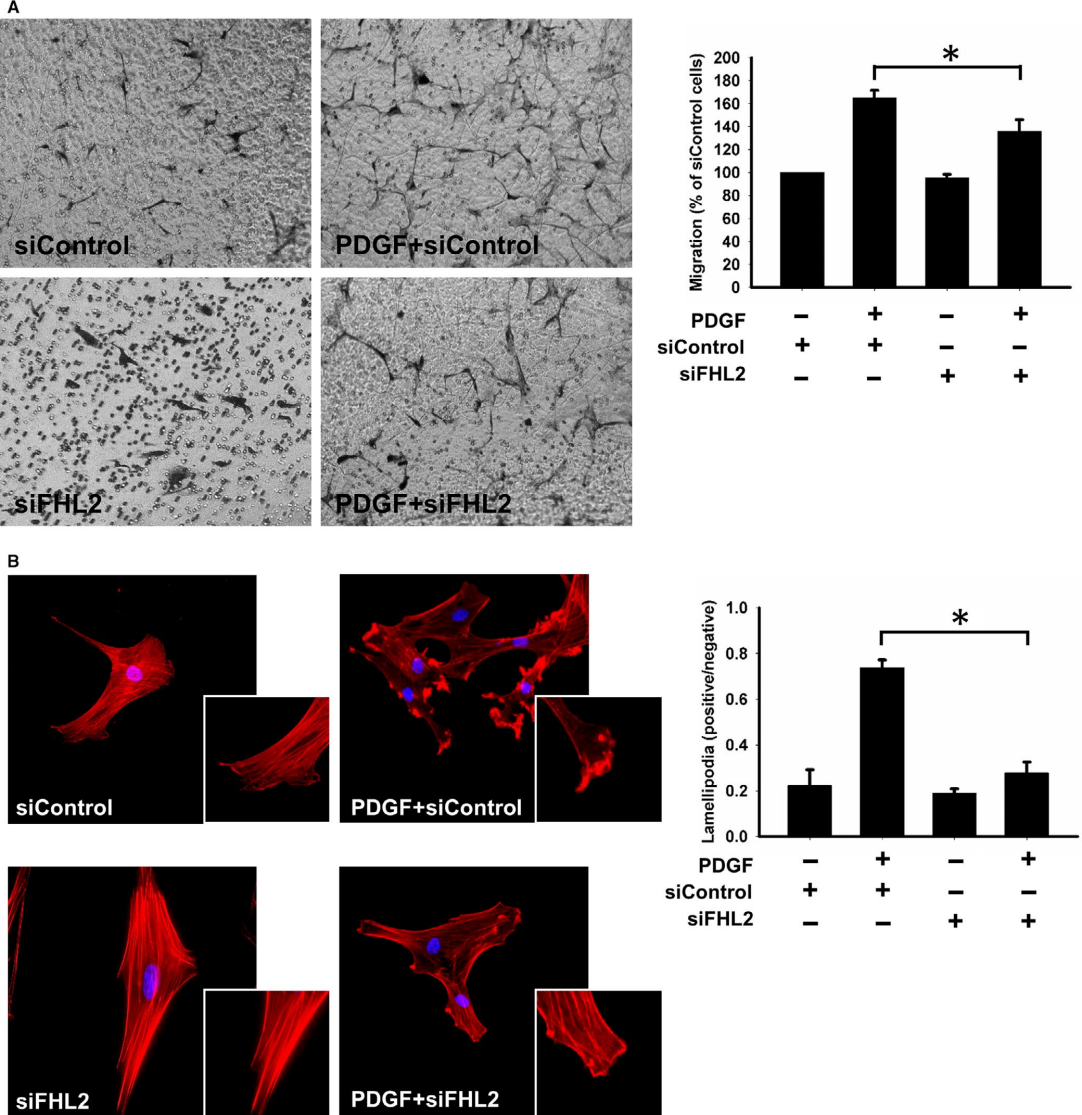
Effects of FHL2 deficiency in migration of HASMCs. FHL2 deficiency modulated HASMCs migration and cytoskeleton conformational changes. A, Effect of FHL2 deficiency on the migration of HASMCs. Cells were transfected with FHL2 siRNA for 24 h and placed onto the upper side of a transwell towards PDGF (10 ng/mL PDGF located in the lower chamber). The histogram showed the percentage of migrating cells. B, The effect of FHL2 deficiency on the formation of lamellipodia was induced by PDGF in HASMCs. After 15 min of PDGF treatment, HASMCs were stained with F‐actin and DAPI. These results are expressed as the mean ± SEM of five separate experiments run in triplicate. (*P < .01)

Next, we examined the role of FHL2 in PDGF‐induced HASMC migration. As expected, PDGF promoted the migration of HASMCs in the modified Boyden chamber. FHL2 silencing partly inhibits PDGF‐induced migration (n = 5; Figure 6A). Therefore, these data suggest that FHL2 is involved in PDGF‐induced HASMC migration.

Migrating cells are polarized with membrane protrusions at the leading edge. The protrusions with lamellipodial growths form new adhesions and play crucial roles in cell migration. To assess the role of FHL2 in the establishment of lamellipodia, lamellipodia formation with positive F‐actin expression in HASMCs was evaluated using immunocytochemistry. Without a PDGF stimulus, F‐actin was maintained at the cell membrane, and lamellipodia were not formed in >80% of HASMCs. In contrast, the number of lamellipodia accompanied by F‐actin increased in PDGF‐stimulated HASMCs (n = 5; Figure 6B). This effect was not observed in FHL2‐silenced HASMCs, suggesting that the effect of PDGF on lamellipodia formation is mediated by FHL2.

**Figure 7:**
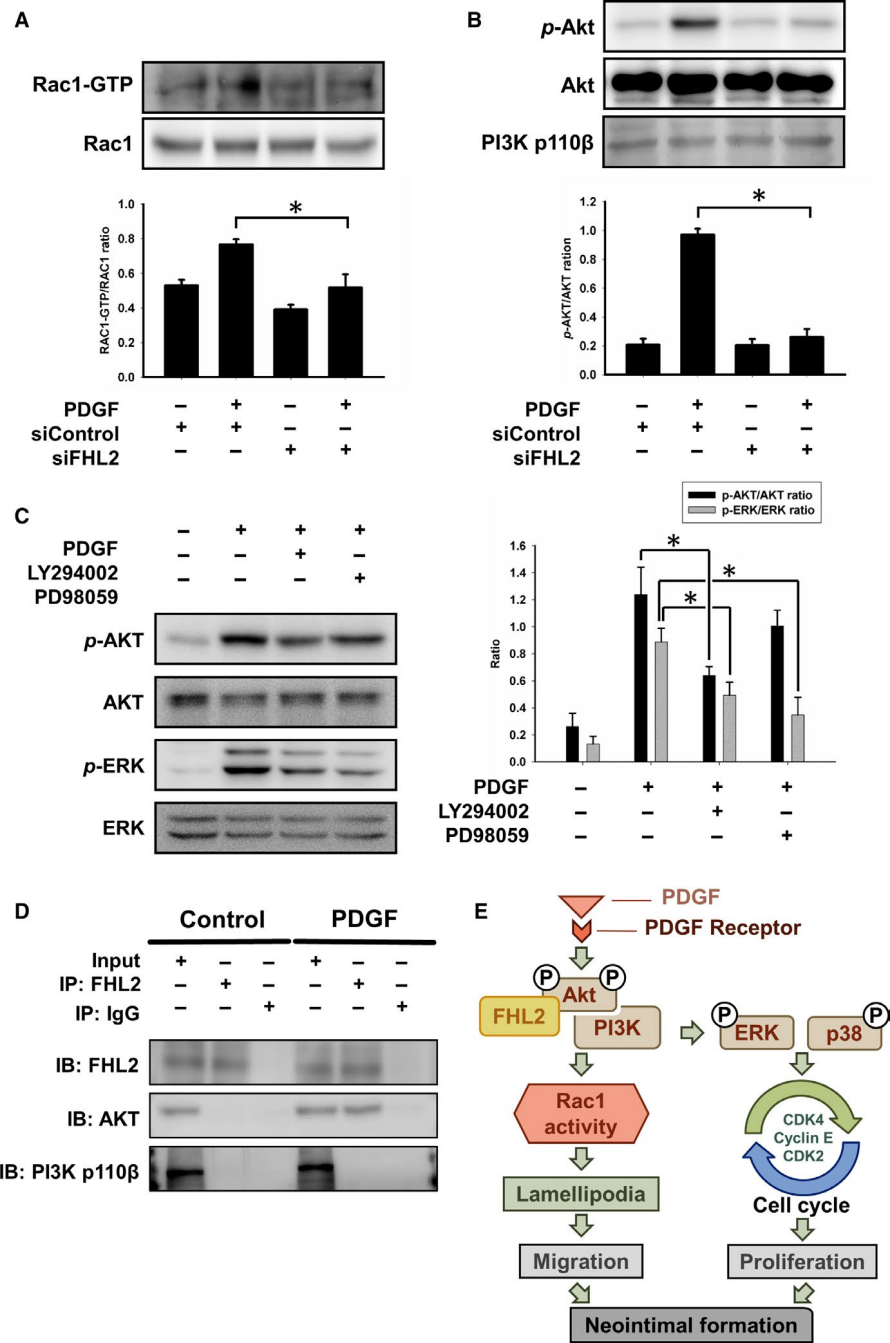
FHL2 deficiency modulated Rac1 activation and related signalling pathway. A, Cell extracts were harvested after 24 h transfection and proceeded the Rac1 pull‐down assay. FHL2 siRNA down‐regulated the levels of activated Rac1. B, Cells were transfected with FHL2 siRNA for 24 h and calculated the ratio of AKT phosphorylation after PDGF treatment using Western blotting. C, Cells were stimulated in PDGF for 15 min after pre‐treated with Akt inhibitor (LY294002) and ERK inhibitor (PD98059) for 1 h. Western blot showed the expression of Akt and ERK phosphorylation. D, PDGF treated transfected cells for 15 min. Co‐immunoprecipitation revealed the interaction of FHL2 and AKT. E, Proposed scheme of HASMCs antiproliferation and antimigration by FHL2 through Rac1‐AKT and MAPK pathways. FHL2 directly controlled AKT phosphorylation. FHL2 deficiency led to Rac1 inactivation and cell cycle arrest. FHL2 plays a pivotal role in the AKT and MAPK signalling pathway in neointimal formation. These results are expressed as the mean ± SEM of five separate experiments run in triplicate. (*P < .01)

Rac1‐GTP activity is pivotal for cytoskeleton conformational changes. Based on the above findings, we examined the involvement of FHL2 and the activity of Rac1 in lamellipodia formation. FHL2 silencing significantly reduced the PDGF‐induced Rac1‐GTP expression ratio from 0.7855 ± 0.0183 to 0.4945 ± 0.0257 (n = 5; Figure 7A).

### 3.7 FHL2 interacts with AKT as a scaffold protein and regulates phosphorylation

Knockdown of FHL2 regulated cytoskeleton conformational changes. The AKT‐Rac1 signalling pathway is critical for cell structure remodelling. FHL2 silencing prevented PDGF‐induced AKT phosphorylation (n = 5; Figure 7B). According to previous study, we hypothesize PDGF‐stimulated AKT phosphorylation regulated ERK phosphorylation. These results showed that inhibition of AKT significantly decreased the phosphorylation of ERK. However, inhibition of ERK did not significantly decrease the AKT phosphorylation. (n = 5; Figure 7C).

In previous studies, FHL2 was considered to be a scaffold protein.^18^ To investigate whether FHL2 regulating PI3K/AKT signalling pathway by protein‐protein interaction. We performed co‐immunoprecipitation to reveal that FHL2 interacted with AKT but not PI3K. The expression of AKT was detected in HASMCs following PDGF stimulation (n = 5; Figure 7D). These findings reveal that FHL2 interacts with AKT.

## 4 DISCUSSION

In the present study, we identified FHL2 as a novel regulator of HASMC proliferation and migration as well as HASMC‐mediated vascular remodelling. FHL2 is barely detectable in normal HASMCs. However, in response to PDGF, which is up‐regulated by restricted blood flow‐induced atherosclerosis, FHL2 is induced during HASMC proliferation and migration and interacts with AKT, subsequently modulating ERK1/2 and p38 phosphorylation.

Vascular injury triggers VSMC activation in the media and an inflammatory response.^19^ Absence of FHL2 reduced the atherosclerotic plaque formation by regulating the inflammatory process including macrophage and monocyte recruitment and anti‐inflammatory cytokine secretion.^13^ The activated VSMCs undergo migration, proliferation and apoptosis.^20^ A previous study showed that FHL2 deficiency with complete artery ligation enhanced neointimal formation by ERK phosphorylation and cyclin D1 increase.^14^ Complete artery ligation might not be a physiologically relevant model because of the lack of blood flow, endothelial denudation and thrombosis.^21^ Thrombi released TGF‐β1 in response to vascular injury in the carotid artery of mice.^21^ A high concentration of TGF‐β1 might stimulate VSMC proliferation and neointimal formation.^22^ ELISA showed that the active form of the TGF‐β1 in Kupffer cells from FHL2‐deficient mice was expressed at significantly higher levels than that in wild‐type mice. The deletion of FHL2 enhanced TGF‐β1 expression and the luciferase reporter assay showed that FHL2 negatively regulated the TGF‐β1 promoter in wild‐type and FHL2‐deficient embryonic fibroblasts.^23^ Instead, we used a partial ligation model in vivo and human artery smooth muscle cell in vitro to recapitulate the disease process. We speculate that the paradoxical results were due to locally high concentrations of TGF‐β1 released from complete artery ligation‐induced thrombi formation in FHL2‐deficient mice, which resulted in neointimal formation. Vascular smooth muscle cell migration is an important process during neointimal hyperplasia. Ligation‐induced vascular injury increases the serum levels of several cytokines, such as PDGF.^24^ We found that knockdown of FHL2 reduced the phosphorylation of PDGF receptor. We suggested that FHL2 deficiency attenuated the PDGF secretion contributed to the down‐regulation of PDGF receptor phosphorylation (Figure 3D). These cytokines trigger VSMC cytoskeleton conformational changes and lamellipodia formation. Platelet‐derived growth factor is a strong mitogenic factor for smooth muscle cell proliferation.^25^ Secretion of PDGF activates several signalling pathways, such as MAPK and PI3K/AKT. Unlike the vascular endothelial cell growth factor/FHL2 signalling pathways in CPCs,^26^ PDGF induces VSMC proliferation via AKT. Vascular smooth muscle cell migration from the media to the intima and subsequent proliferation are important for the pathogenesis of vascular diseases, such as atherosclerosis,^27^ neointimal hyperplasia^24^, ^28^ and calcification.^29^ AKT phosphorylation plays a major role in these processes. The phosphorylation of AKT is modulated by the level of PI3K activation or phosphatase and tensin homologue (PTEN) dephosphorylation.^30^ Moreover, FHL2 participates in gene transcription as a cofactor. FHL2 interacted with the myocardin family of SRF and increased SMC‐specific promoter activity by protecting myocardin degradation.^11^

Modulation of the VSMC phenotype is essential for vascular haemostasis. In response to vascular injury, VSMC phenotypic switching is a crucial process for vessel repair. Vascular smooth muscle cells go from a contractile state to a synthetic state in which their proliferative ability is increased.^1^ A previous study showed that FHL2 is a regulator that interacts with BMPRII during the induction of the contractile state. Instead of inhibiting recruitment of SRF/MRTF‐A, FHL2 inhibited the recruitment of SWI/SNF complex protein Brg1 and RNA polymerase II.^12^ Our study demonstrated that FHL2 is critical for the PDGF‐mediated up‐regulation of AKT phosphorylation, Rac1 activation and lamellipodia formation in VSMCs. These results demonstrate that FHL2 promotes VSMC proliferation and migration, leading to vascular remodelling/neointimal hyperplasia. Importantly, vascular injury induces FHL2 expression in medial VSMCs and neointimal VSMCs. After CAL, VSMCs respond to injury‐induced serum factors, such as PDGF. The initial expression of FHL2 in medial VSMCs is likely attributed to VSMC proliferation in the injured arteries. The reduced intimal hyperplasia and decreased activation of proliferative signalling pathways in ligation‐injured FHL2^−/−^ mouse arteries suggest that FHL2 plays a role in vascular remodelling in vivo. Our previous study demonstrated that FHL2 deficiency contributes to circulating proangiogenic cell (CPC) dysfunction and the inhibition of cytokine secretion.^26^ However, in this study, we observed VSMC dysfunction and cell cycle arrest after the knockdown of FHL2.

FHL2 knockdown resulted in the down‐regulation of AKT phosphorylation. Neointimal formation is reduced following the suppression of PI3K,^31^ whereas neointimal hyperplasia is enhanced following the suppression of PTEN.^32^ Previous studies have shown that the activation of PI3K/AKT signalling promotes VSMC proliferation and migration.^33^ In this study, we found that FHL2 contributed to neointima formation by regulating VSMC migration and proliferation. High levels of FHL2 expression could be detected after CAL, whereas an FHL2 deficiency in VSMCs led to significantly reduced neointima formation. FHL2 forms a complex with PI3K and AKT, leading to AKT phosphorylation and VSMC migration.

Protein kinase signalling pathways are involved in VSMC proliferation and survival. The MAPK and PI3K/AKT pathways play key roles in cell proliferation and other cellular processes. Our data were compatible with previous studies that the AKT pathway is correlated with the MAPK signalling pathway (Figure 7C); for example, ERK1/2 inactivation inhibits human VSMC proliferation^5^, ^34^, ^35^, ^36^ and p38 protein kinase activation.^37^, ^38^ The absence of FHL2 modulates VSMC proliferation by regulating the MAPK signalling pathway via the inhibition of MEK/ERK phosphorylation. Our data showed that down‐regulation of FHL2 regulated the AKT and MAPK signalling pathway, but these findings should be confirmed in the tissue sections in the following research. Cell cycle progression is also essential for cell proliferation. Cyclins and CDKs are positive regulators that are directly involved in cell proliferation. CDKIs, such as p21 and p27, suppress CDK activation and cause cell cycle arrest.^5^ Previous studies have shown that p27 binding to CDK2 inhibits VSMC cycle progression.^39^ Decreased expression of cyclin D and CDKs induces G1 arrest, which also inhibits VSMC proliferation.^40^ Furthermore, the suppression of p27 expression affects the mitotic cell cycle in VSMCs.^41^ We observed that knockdown of FHL2 inhibited VSMC entry into the S phase by decreasing cyclin D/E and CDK2/4 levels, and increasing the level of p27. p38‐MAPK inhibits cyclin D1 expression and degrades cyclin D3.^17^, ^42^ In addition to its effect on proliferation, p38‐MAPK activation causes growth arrest by inducing apoptosis in various cell types, including VSMCs.^43^, ^44^ Consistent with previous findings, the absence of FHL2 causes cell cycle arrest via the activation of p38 phosphorylation.^17^

Unlike skeletal cells, cardiac muscle cells and other cells, VSMCs retain the ability to differentiate.^45^ VSMCs are able to transdifferentiate into a synthetic phenotype by decreasing VSMC marker expression, increasing matrix deposition and activating cell migration and growth signals.^1^ Reports have shown that FHL2 regulates the assembly of extracellular matrix proteins^46^ and interacts with focal adhesion kinase in epithelial ovarian cancer cells^47^ and ERK 2 in cardiomyocytes.^48^ Rac1‐GTP activation plays an important role in cytoskeleton conformational changes. Previous studies have shown that FHL2 interacts with sphingosine kinase‐1 (SK‐1) and inhibits AKT/PI3K activation in ECs. Finally, activated‐SK1 promotes Gα‐linked Rho‐ and Rac GTPase‐dependent cytoskeletal rearrangements.^49^ Rac1 promotes cell proliferation and migration in several ways. Rac1 promotes the progression of the G1/S phase in the cell cycle by increasing the expression of cyclin D1.^50^ Previous studies have revealed that decreased expression of Rac1 arrests cells in the G2/M phase, which indicated the role of Rac1 in cell mitosis. Interestingly, accumulation of activated Rac1 in the nucleus in the late G2 phase increases the mitotic rate.^50^, ^51^ Our study revealed that PDGF‐induced the phosphorylation of AKT and activation of Rac1‐GTP. Down‐regulation of FHL2 decreased AKT phosphorylation, inhibited Rac1‐GTP activation and inhibited lamellipodia formation. Finally, down‐regulation of FHL2 inhibited the directional migration of VSMCs towards the source of PDGF. According to the previous study, PI3K phosphorylated AKT with PDGF stimulation. Our findings revealed that there was no significant change in the protein expression level of PI3K p110β. PI3K was composed of a catalytic and regulatory subunit. However, further studies are needed to clarify how FHL2 protein regulates PI3K activity. In this study, we identified the molecule signalling regulation and cell structure change in HASMCs, but VSMCs isolated from wild‐type and FHL2 deficient mice might be more reliable in the current study.

Taken together, our study identified FHL2 as a novel regulator of VSMC proliferation and migrative modulation as well as VSMC‐mediated vascular remodelling. Targeting FHL2 might be an effective approach in treating proliferative vascular disorders.^52^, ^53^, ^54^, ^55^

## CONFLICT OF INTEREST

The authors confirm that there are no conflicts of interest.

## AUTHOR CONTRIBUTIONS

Chen CY, Tsai SY, Tsai SH, Chu PH, Huang PH, Chen JW, & Lin SJ participated in research conception and design. Chen CY, Tsai SY, & Tsai SH involved in data analysis and interpretation. Chen CY, Tsai SY, & Tsai SH performed statistical analysis. Chen CY, Tsai SH, & Huang PH carried out drafting of the manuscript. All authors contributed to the critical revision and approval of final manuscript.

## ETHICAL APPROVAL

The animal experiment was approved and monitored by the Institutional Animal Care and Use Committee of Taipei Veterans General Hospital (IACUC no. 2014‐221, Taipei, Taiwan) and complied with the Guide for the Care and Use of Laboratory Animals published by the US National Institute of Health (8th edition, 2011).

## Data Availability

The data that support the findings of this study are available from the corresponding author on reasonable request.
